# Sodium-Glucose Cotransporter-2 Inhibitors Ameliorate Liver Enzyme Abnormalities in Korean Patients With Type 2 Diabetes Mellitus and Nonalcoholic Fatty Liver Disease

**DOI:** 10.3389/fendo.2021.613389

**Published:** 2021-06-10

**Authors:** Won Euh, Soo Lim, Jin-Wook Kim

**Affiliations:** ^1^ Department of Internal Medicine, Seoul National University College of Medicine, Seoul, South Korea; ^2^ Department of Internal Medicine, Seoul National University Bundang Hospital, Seongnam, South Korea

**Keywords:** type 2 diabetes mellitus, sodium-glucose cotransporter 2 (SGLT2) inhibitor, alanine aminotransferase, body weight, propensity score (PS) matching (PSM)

## Abstract

Sodium-glucose cotransporter-2 inhibitors (SGLT2is) are reported to reduce body fat in patients with type 2 diabetes mellitus (T2DM), and SGLT2i-induced weight reduction may help improve comorbid nonalcoholic fatty liver disease (NAFLD). This study aimed to investigate the potential benefit of SGLT2is over other oral antidiabetic drugs (OADs) in patients with T2DM-associated NAFLD. We enrolled real-world Korean patients with T2DM-associated NAFLD in whom initial metformin therapy had been modified by stepwise addition of OAD(s) due to insufficient glucose control. Propensity score (PS) matching was used for the comparison of changes in clinical and biochemical parameters to balance potential covariates. Among the 765 enrolled patients, 663 patients received additional OADs other than SGLT2i and 102 patients received SGLT2i therapy. PS matching selected 150 and 100 patients from the control and the SGLT2i group, respectively. The SGLT2i group lost more weight than the control group at 6 months (mean –1.3 kg *vs.* 0.0 kg; *P* < 0.001). Alanine aminotransferase (ALT) levels also decreased more in the SGLT2i group at 3 (–11 U/L *vs.* –1 U/L), 6 (–12 U/L *vs.* –1 U/L), and 12 months (–14 U/L *vs.* –2 U/L) (all *P* < 0.05). Addition of SGLT2is was an independent predictor of ALT improvement in a multivariate logistic regression model (odds ratio 1.91; *P* = 0.016). Compared with other OADs, addition of SGLT2is was more effective in weight reduction and ALT improvement in patients with T2DM and comorbid NAFLD.

## Introduction

Nonalcoholic fatty liver disease (NAFLD) is a common comorbidity of type 2 diabetes mellitus (T2DM) and one half to two thirds of T2DM patients have NAFLD (1). Close association between T2DM and NAFLD is related to the fact that metabolic syndrome is a common risk factor for both conditions, frequently associated with adiposity and insulin resistance (2, 3).

The shared disease pathogenesis between T2DM and NAFLD raised the possibility of the potential role of antidiabetic drugs in NAFLD management. There have been several studies which investigated the effects of oral antidiabetic drugs (OADs) such as metformin (4), thiazolidinediones (5, 6) and dipeptidyl peptidase 4 inhibitors (DPP4is) (7) on NAFLD. Although some studies reported histologic improvements in NAFLD, the clinical benefit of OADs lack sufficient evidence for them to be routinely recommended in NAFLD treatment (8, 9).

Sodium-glucose cotransporter 2 inhibitors (SGLT2is) decrease blood sugar levels by increasing urinary glucose excretion. Interestingly, SGLT2is are shown to decrease total body and visceral fat masses in patients with T2DM (10–12). Since weight reduction is the main strategy for decreasing hepatic steatosis, it may be postulated that SGLT2is have potential for the management of T2DM-associated NAFLD. In a recent phase 4 study (n = 84), empagliflozin 25 mg/day for 24 weeks reduced liver fat content by 22% as measured by magnetic resonance imaging (MRI) in European patients with T2DM (13). Notably, the addition of 10 mg empagliflozin daily led to a significant reduction in the MRI proton density fat fraction (MRI-PDFF), a useful quantitative indicator of the liver fat content, from 16.2% to 11.3% at 20 weeks in Indian patients with T2DM (n = 50) (14). After treatment for 24 weeks with 2.5 mg luseogliflozin, another SGLT2i, the MRI-PDFF was reduced significantly from 21.5 ± 7.2% to 15.7 ± 6.8% in Japanese patients with T2DM (n = 40) (15). In another European study (n = 32), treatment with dapagliflozin 10 mg/day for 8 weeks significantly decreased liver MRI-PDFF by 13% in patients with T2DM (16). These findings suggest that SGLT2is may have additional benefit of improving steatosis in T2DM-associated NAFLD.

Since alanine aminotransferase (ALT) is commonly used to measure liver injury, ALT has been used as an endpoint marker in many NAFLD and nonalcoholic steatohepatitis (NASH) studies. Several observational studies and recent small, controlled trials also reported that SGLT2i therapy improved ALT levels in T2DM-associated NAFLD (17–19). A recent meta-analysis reported that SGLT2is could reduce the level of ALT, an easily accessible serum marker of hepatic steatosis, although heterogeneity of the studies included in this analysis was substantial (I^2^ = 73%) (20). Thus, the effect of SGLT2i on liver enzyme levels is yet to be confirmed in larger studies.

In this study, we aimed to investigate the benefit of SGLT2is over other OADs, especially effect on serum transaminase levels, in a real-world practice of T2DM-associated NAFLD. For this purpose, we assessed changes in serum transaminase levels as an endpoint in a propensity score (PS) matched cohort of T2DM patients with comorbid NAFLD.

## Methods

### Study Design

This single-center retrospective cohort study recruited consecutive patients with T2DM and NAFLD who visited Seoul National University Bundang Hospital (SNUBH), a tertiary medical center in South Korea, between September 2014 and September 2018. An electronic cohort was established retrospectively by using the electronic medical record system of SNUBH (21). The eligibility criteria were as follows: patient age >18 years, presence of fatty liver by ultrasound, and at least 3 months of metformin-alone therapy for T2DM followed by stepwise addition of OADs and maintenance for at least 3 months. The exclusion criteria were as follows: treatment with a glucagon like peptide-1 receptor agonist (GLP-1 RA) or insulin within 6 months from the initial metformin therapy, chronic liver diseases other than NAFLD (e.g., viral hepatitis, autoimmune liver disease and drug-induced liver injury), significant alcohol consumption (daily intake >30 g for men and >20 g for women), or malignancies.

The enrolled patients were classified into either SGLT2i or control groups according to the modification of metformin therapy. The SGLT2i group added one of the available SGLT2is (dapagliflozin, empagliflozin, or ipragliflozin) to metformin. The control group added OAD(s) other than SGLT2is, i.e., DPP4is, sulfonylureas, or thiazolidinedione, to metformin. The choice of additional OADs was at the discretion of the attending physicians. All patients in this study were advised to maintain a healthy lifestyle by being more active in daily life and to avoid consuming a high fat, high carbohydrate diet. Diagnosis of fatty liver was made when ultrasonographic findings showed increased hepatic echogenicity compared with the right renal cortex (22). Hepatic steatosis manifests as increased echogenicity and beam attenuation in the ultrasonographic examination (22). This results in liver parenchyma appearing relatively hyperechoic compared with the renal cortex (normally liver and renal cortex are of a similar echogenicity) (23). Fatty liver also shows hyperechogenicity relative to the spleen (23).

PS matching was used to balance potential covariates between the two groups by matching the following baseline variables as covariates: age, body mass index (BMI), hypertension, dyslipidemia, and circulating glycated hemoglobin (HbA1c) and ALT levels. The PS was calculated from a logistic model, and k-nearest-neighbor matching without replacement and caliper of 0.01 was performed using the “psmatch2” tool of STATA software (version 14, STATA Corporation, College Station, TX, USA).

This study was performed in accordance with the *Ethical Principles for Medical Research Involving Human Subjects* outlined in the Declaration of Helsinki in 1975 (revised in 2013; https://www.wma.net/policies-post/wma-declaration-of-helsinki-ethical-principles-for-medical-research-involving-human-subjects/). The institutional review board of SNUBH reviewed the study protocols and case report form and approved this study (IRB No: B-1810/497-007). Informed consent was waived because of the retrospective nature of the study and anonymous nature of the clinical data according to the by Guideline for Good Clinical Practice of International Council for Harmonization of Technical Requirements for Registration of Pharmaceutical for Human Use (E6R2).

### Assessment of Anthropometric Parameters

Clinical parameters, including blood pressure, body weight, and BMI, were measured using standard methods. The BMI was calculated by dividing the subject’s weight (kg) by height squared (m^2^). Systolic and diastolic blood pressures (SBP and DBP, respectively) were measured with subjects in a seated position using an electronic blood pressure meter (UA-1020 device; A&D Co., Tokyo, Japan). Blood pressure was measured twice 5 min apart and the mean value was used in the analysis.

### Measurement of Biochemical Factors

Blood sampling was carried out after a 10-h overnight fast. The samples were centrifuged immediately at 3,000 rpm for 10 min at 4°C. HbA1c level was measured using a Bio-Rad Variant II Turbo HPLC analyzer (Bio-Rad, Hercules, CA, USA) in SNUBH, the National Glycohemoglobin Standardization Program level II certified laboratory. Fasting plasma glucose (FPG) levels were analyzed using the hexokinase method. Triglyceride (TG) levels were measured by the glycerol-3-phosphate oxidase peroxide method, and high-density lipoprotein (HDL)- and low-density lipoprotein (LDL)-cholesterol were measured by homogeneous enzymatic assays. ALT and aspartate aminotransferase (AST) levels were measured using the NADH-UV method. Hepatic steatosis index (HSI) was calculated: HSI = 8 × ALT/AST ratio + BMI (+2, if diabetes mellitus; +2, if female) (24). Serum creatinine (Cr) was measured by Jaffe’s kinetic method using a Hitachi 747 chemistry analyzer (Hitachi, Tokyo, Japan). The Chronic Kidney Disease Epidemiology Collaboration (EPI) equations were employed to derive the estimated glomerular filtration rate (eGFR).

### Efficacy Assessment

The primary endpoint was a change in the ALT concentration from baseline. Secondary outcomes included changes in FPG and HbA1c levels, changes in AST, and changes in lipid profiles. Renal function was also assessed. The relationship between changes in ALT levels and pre-specified parameters such as age, sex, and BMI was also investigated.

### Statistical Analysis

Clinical and biochemical parameters were compared using Student’s *t* tests for continuous variables and chi-square tests for categorical variables. The Mann–Whitney nonparametric *U* test and χ^2^ test were used for the assessment of continuous and categorical variables, respectively. Subgroup analysis was performed to identify subsets of patients who would be more likely to benefit from therapy with SGLT2is. The “ipdover” tool of STATA was used to generate data for forest plots outside the context of meta-analysis, without pooling or heterogeneity testing. Ipdover creates forest plots of subgroup analyses within one trial dataset. A *p* value < 0.05 was considered to be significant.

## Results

### Baseline Characteristics Before and After PS Matching

**Figure 1:**
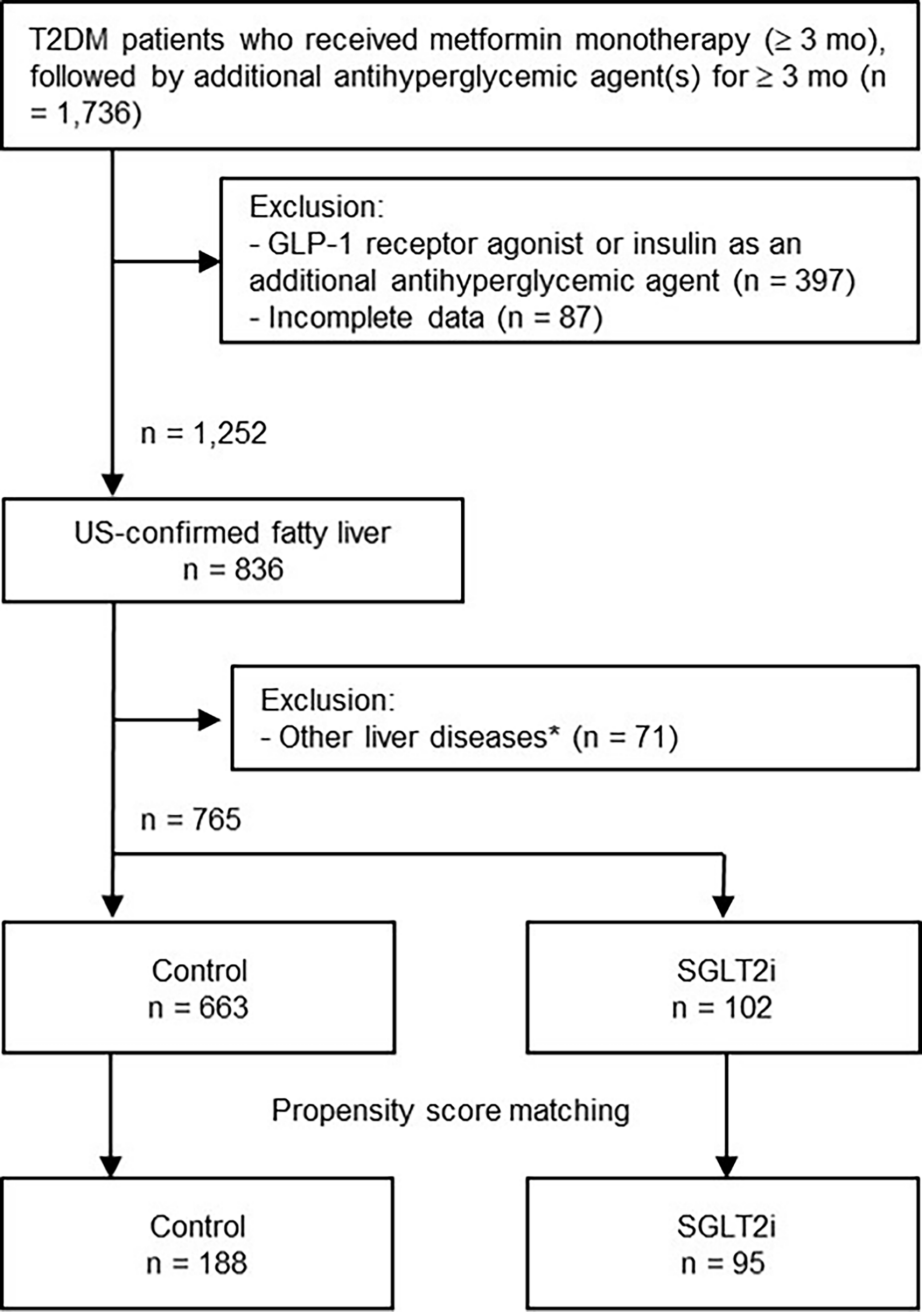
Flowchart of the selection process. *Other liver diseases included viral hepatitis B and C, autoimmune hepatitis, drug-induced liver injury, and history of excessive alcohol intake (daily intake > 30 g for men and > 20 g for women). GLP-1, glucagon-like peptide-1; US, ultrasound; SGLT2i, sodium-glucose cotransporter-2 inhibitor.

The selection process of the study population is shown in **Figure 1**. A total of 1,736 records of patients with T2DM were retrieved from the databank, who had received initial metformin-based therapy for at least 3 months and subsequent additional OAD(s) for ≥3 months. After excluding 971 patients due to use of insulin or GLP-1 RA (n = 397), missing laboratory data (n = 87), a lack of liver ultrasound data (n = 416), and other liver diseases such as viral hepatitis, autoimmune liver diseases and drug-induced liver injury (n = 71), 765 patients with T2DM and NAFLD were finally enrolled.

The baseline characteristics of the study population are presented in **Table 1**. Before PS matching, the SGLT2i group (n = 102) was significantly younger and had higher body weight and BMI compared with the non-SGLT2i control group (n = 663). The HSI was significantly higher in the SGLT2i group (24). The prevalence of hypertension, triglyceride levels, HbA1c and ALT levels were also significantly different between the two groups. The PS matching procedure selected 95 patients from the SGLT2i group and 188 from the control group. After PS matching, the two groups were balanced for all parameters, including HSI.

**Table 1:** Baseline characteristics of the study population.

	Original cohort			Propensity score-matched cohort		
	Control	SGLT2i	*P*	Control	SGLT2i	*P*
	*n=*663	*n=*102		*n=*188	*n=*95	
Age (years)	63 (18)	54 (17)	<0.001	58 (17)	56 (14)	0.138
Male gender	338 (52%)	63 (62%)	0.080	112 (60%)	58 (61%)	0.811
Body weight (kg)	69.0 (15.7)	76.5 (22.4)	<0.001	72.7 (16.4)	73.0 (22.2)	0.240
BMI (kg/m^2^)	25.7 (3.9)	27.7 (5.3)	<0.001	26.7 (4.2)	27.5 (5.0)	0.229
Fasting glucose (mg/dL)	146 (41)	145 (38)	0.256	130 (38)	125 (35)	0.105
HbA1c (%)	7.4 (0.9)	7.3 (1.0)	0.007	7.4 (0.8)	7.3 (1.1)	0.438
Total cholesterol (mg/dL)	165 (35)	170 (34)	0.201	159 (55)	162 (49)	0.376
Triglyceride (mg/dL)	134 (83)	155 (104)	0.006	140 (76)	147 (93)	0.135
HDL-cholesterol (mg/dL)	49 (10)	50 (10)	0.482	47 (12)	49 (12)	0.220
LDL-cholesterol (mg/dL)	90 (38)	90 (34)	0.926	89 (39)	87 (35)	0.889
Serum creatinine (mg/dL)	0.8 (0.2)	0.8 (0.2)	0.395	0.8 (0.3)	0.8 (0.4)	0.394
eGFR-EPI (mL/min/1.73 m^2^)	91 (21)	99 (21)	<0.001	95 (16)	99 (29)	0.075
AST (U/L)	27 (25)	29 (15)	0.501	29 (18)	29 (17)	0.908
ALT (U/L)	26 (25)	36 (31)	0.021	30 (37)	35 (31)	0.809
γGT (U/L)	56 (65)	54 (68)	0.768	37 (48)	32 (28)	0.319
Hepatic steatosis index*	36.8 (7.2)	40.1 (10.1)	< 0.001	37.9 (8.5)	39.4 (7.1)	0.409
*Comorbidity*						
Hypertension (%)	343 (52%)	42 (41%)	0.047	89 (47%)	41 (43%)	0.505
Dyslipidemia (%)	324 (49%)	52 (51%)	0.691	99 (53%)	48 (51%)	0.734
Statin usage (%)	458 (69%)	75 (74%)	0.363	134 (71%)	73 (77%)	0.318
Liver cirrhosis (%)	28 (4%)	1 (1%)	0.110	2 (1%)	0 (0%)	0.313

Continuous variables are expressed as the median (interquartile range) and categorical variables are expressed as the number (percentage). P values were calculated by using Mann–Whitney nonparametric U test and χ^2^ test for continuous and categorical variables, respectively. BMI, body mass index; ALT, alanine aminotransferase; AST, aspartate aminotransferase; γGT, γ-glutamyl transferase; HbA1c, glycated hemoglobin; HDL, high-density lipoprotein; LDL, low-density lipoprotein.

*Hepatic steatosis index = 8 × ALT/AST + BMI (+2 if diabetes mellitus yes, +2 if female) (24).

Among other OADs, DPP4is were the most common agent used (n = 160), followed by sulfonylureas (n = 23) and thiazolidinedione (n = 5). Among the SGLT2is, dapagliflozin was most frequently used (n = 58), followed by empagliflozin (n = 34) and ipragliflozin (n = 3).

### Changes in Body Weight, FPG, HbA1c, Lipid Profiles, and Transaminases by SGLT2is

**Table 2** shows the changes in body weight and laboratory values at 6 months after the addition of OADs to metformin. Body weight reduced more in the PS-matched SGLT2i group (mean –2.5 kg, 95% confidence interval [CI]: –0.6 to –4.5) compared with controls (mean –0.2 kg, 95% CI: 0.2 to –0.5) (*P* = 0.001). Changes in HbA1c and lipid levels were similar between the two groups, whereas glomerular filtration rate increased significantly in the PS-matched SGLT2i group. Changes in AST levels were similar between the two groups, whereas ALT levels decreased more in the PS-matched SGLT2i group than in controls 6 months (–13 U/L *vs.* –5 U/L; *P* = 0.033) and 9 months (–15 U/L *vs.* –5 U/L; *P* = 0.014; **Table 3**). The HSI also decreased significantly more at 6 months in the SGLT2i group (–1.1 *vs.* 0.2, *P* = 0.005; **Table 3**). When the comparison was limited to DPP4i *vs.* SGLT2i, ALT responses were still better in the SGLT2i group (**Supplementary Table 1**).

**Table 2:** Comparison of 6-month changes in body weight, serum glucose, and lipid profiles between the propensity score-matched control and SGLT2i groups.

	Control	SGLT2i	*P*
	*n=*188	*n=*95	
ΔWeight (kg)	-0.2 (–0.5, 0.2)	–2.5 (–4.5, –0.6)	0.001
ΔHbA1c (%)	–0.5 (–0.6, –0.4)	–0.6 (–0.8, –0.4)	0.273
ΔFasting glucose (mg/dL)	–14 (–20, –8)	–21 (–26, –15)	0.130
ΔTriglyceride (mg/dL)	–11 (–20, -1)	–16 (–28, –4)	0.510
ΔHDL-cholesterol (mg/dL)	0 (–1, 1)	1 (0, 2)	0.267
ΔLDL-cholesterol (mg/dL)	–1 (–4, 2)	–4 (–7, 0)	0.309
ΔeGFR (mL/min/1.73 m^2^)	–1.6 (–3.0, –0.3)	1.2 (–1.4, 3.9)	0.035

Δ: changes at 6 months from baseline values, expressed as the mean and (95% confidence interval). P values were calculated using Student’s t-test. HbA1c, glycated hemoglobin; HDL, high-density lipoprotein; LDL, low-density lipoprotein.

**Table 3:** Comparison of changes in transaminase levels and hepatic steatosis index between the propensity score-matched control and SGLT2i groups.

	Control	SGLT2i	*P*
	*n=*188	*n=*95	
AST changes (IU/L)			
3 months	–2 (–5, 0)	–7 (–12, –1)	0.109
6 months	–4 (–7, –1)	–7 (–13, –1)	0.363
9 months	–4 (–7, 0)	–8 (–14, –2)	0.135
ALT changes (IU/L)			
3 months	–4 (–8, 0)	–11 (–18, –4)	0.063
6 months	–5 (–9, 0)	–13 (–20, –6)	0.033
9 months	–5 (–9, 0)	–15 (–22, –7)	0.014
HSI change at 6 months	0.2 (–0.5, 1.0)	–1.1 (–2.0, 0)	0.005

Values are expressed as the mean (95% confidence interval). P values were calculated using Student’s t-test. ALT, alanine aminotransferase; AST, aspartate aminotransferase; HSI, hepatic steatosis index.

### Predictors of ALT Improvement by SGLT2i Therapy

Next, logistic regression analysis was performed to identify predictors of ALT improvement (>15% reduction from baseline) during the OAD therapy (**Table 4**). Younger age, male sex, body mass index, TG levels and addition of SGLT2is were identified as significant predictors by univariate analysis. In the multivariate analysis, addition of SGLT2is remained significant as a predictor for ALT improvement (odds ratio [OR] 1.73; 95% CI: 1.04–2.92; *P* = 0.036), along with baseline TG levels.

**Table 4:** Logistic regression analyses for predictors of ALT decrease greater than 15% of baseline over 9 months in the propensity score-matched cohort (*n=*283).

Baseline parameter	Univariate analyses			Multivariate analysis		
	OR	95% CI	*P*	OR	95% CI	*P*
Age	0.98	0.96–1.00	0.047	1.00	0.98-1.2	0.995
Male sex	1.63	1.00–2.64	0.049	1.48	0.88–2.49	0.140
Body mass index (kg/m^2^)	1.10	1.03–1.18	0.008	1.07	0.99–1.16	0.076
Fasting glucose (mg/dL)	1.00	0.99–1.01	0.532			
HbA1c (%)	1.06	0.84–1.35	0.623			
Triglyceride (mg/dL)	1.01	1.00–1.01	0.003	1.01	1.00-1.01	0.010
LDL-cholesterol (mg/dL)	1.00	0.99–1.01	0.427			
HDL-cholesterol (mg/dL)	0.99	0.97–1.02	0.681			
SGLT2i *vs.*control	1.78	1.08–2.93	0.023	1.73	1.04–2.92	0.036

OR, odds ratio; CI, confidence interval; other abbreviations as described in **Table 1**.

**Figure 2:**
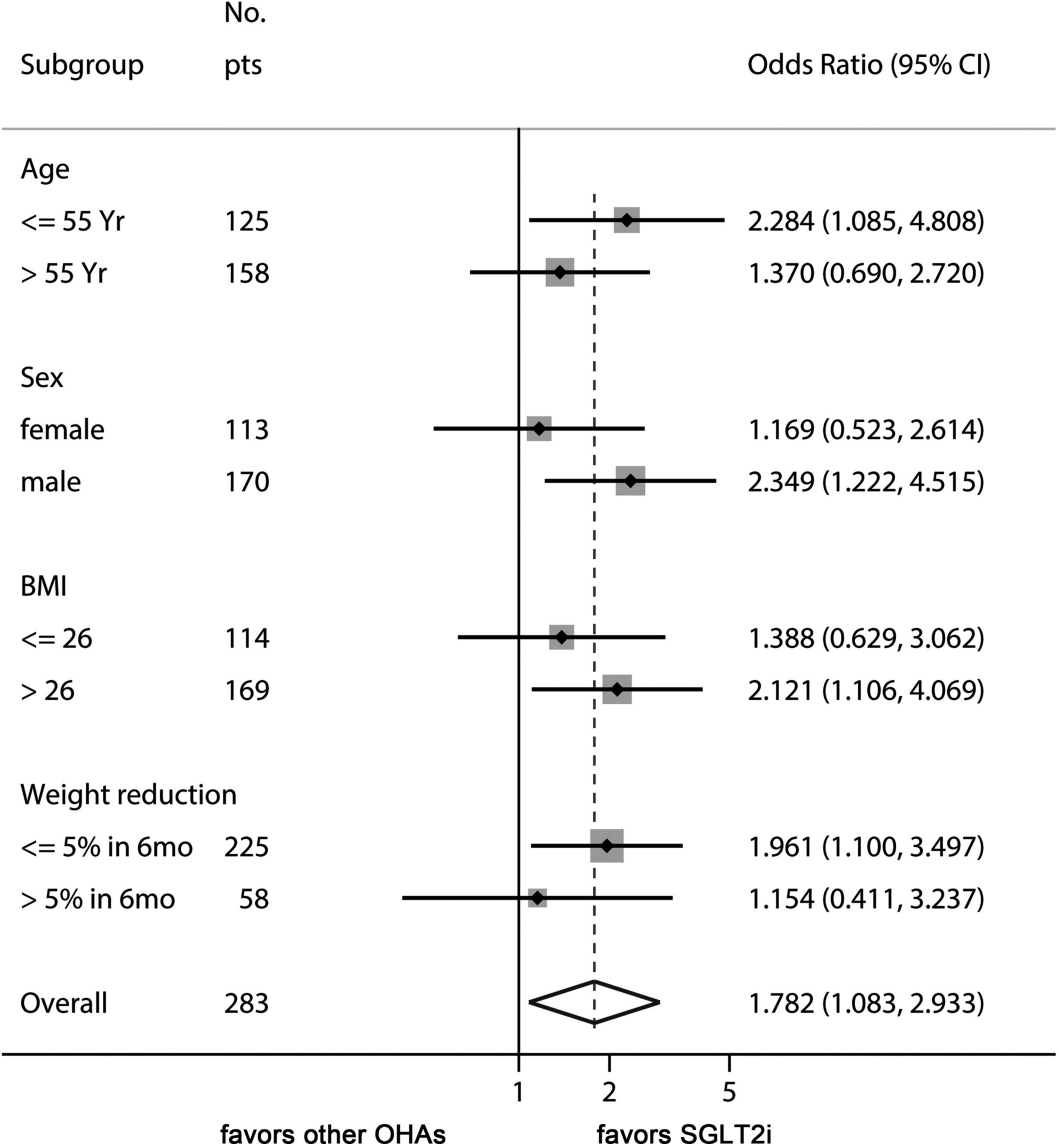
Sensitivity analysis of odds ratio of SGLT2is for managing ALT levels. This Forest plot indicates the odds ratio of SGLT2i over control for the endpoint of ALT decrease ≥ 15% of baseline values. The benefit of SGLT2i for ALT decrease was more prominent in younger patients, male sex, high baseline BMI, and patients with less weight reduction.

Finally, we performed a sensitivity analysis to gain additional insights as to how SGLT2i therapy might improve ALT levels in T2DM-associated NAFLD (**Figure 2**). The beneficial effect of SGLT2is over other OADs was evident in younger patients (<55 years), male patients, patients with baseline BMI >26 kg/m^2^, and patients with less weight reduction during treatment with the study drugs.

## Discussion

Our study showed that addition of SGLT2is induced significantly greater ALT and HSI improvements compared with other OADs in patients with T2DM and ultrasound-confirmed NAFLD on metformin. SGLT2is have emerged as a promising candidate for treating NAFLD because of their potential favorable effect on hepatic fat content in several small studies (14, 25, 26). A retrospective study of 102 Korean patients with T2DM and NAFLD showed that dapagliflozin therapy exhibited greater improvement in liver enzyme levels than DPP4is when used with metformin (27). A few small placebo-controlled trials reported significant decreases in hepatic fat content and/or serum ALT levels by SGLT2i treatment in patients with T2DM and NAFLD (14, 19). However, decreases in ALT were not significantly different in other randomized trials of patients with NAFLD using metformin (26) and pioglitazone (28) as an active control. Inadequate statistical power might have been the reason for these negative results. Our present study included sufficient patient numbers to observe significant changes in ALT levels produced by SGLT2i therapy. Of note, our PS-matching analysis suggests that the use of SGLT2is might confer additional benefits for liver function compared with other OADs among patients with T2DM and NAFLD.

In this study, the HSI was improved by SGLT2i therapy. HSI was derived and validated in a large cohort of >10,000 individuals who underwent health check-ups (24). However, it has a moderate accuracy to detect fatty liver as determined by ultrasonography (24, 29). In a recent prospective cohort study from the Nonalcoholic Steatohepatitis Clinical Research Network (NASH CRN), changes in the ALT levels were significantly associated with fibrosis regression in NAFLD (30).

Reductions in body fat mass and subsequent improvements in insulin resistance might account for the reduction in hepatic fat and ALT levels by SGLT2i therapy. Indeed, addition of SGLT2is significantly reduced body weight compared with other OADs (**Table 2**). Interestingly, the superiority of SGLT2is over other OADs in ALT improvement was observed in patients with less than 5% of weight reduction (**Figure 2**). This result suggests that other mechanisms, such as reduction in inflammatory markers, decreased oxidative stress, and decreased hepatic lipogenesis might have been involved independently of weight reduction (31, 32). Further studies are needed for a mechanistic explanation for the SGLT2i-induced reduction of ALT levels. Being younger and male were also found to be better factors for responding to therapy with SGLT2is in terms of the improvements in liver function.

Small case studies have reported histological improvements in T2DM-associated NAFLD by SGLT2i therapy (33–35). Although our study did not take histological changes into account, previous studies reported associations between ALT improvement and hepatic fat content reduction, as assessed by MRI or computed tomography (14, 19, 26). Because we only assessed ALT changes up to 9 months, long-term hepatic outcomes such as prevention of hepatic fibrosis and hepatic carcinogenesis need to be determined by longer studies.

Current standards of medical care guidelines for T2DM (36, 37) and NAFLD (8) do not recommend specific classes of OADs for T2DM patients with NAFLD, except for pioglitazone in patients with biopsy-proven NASH. Collaborative studies by endocrinologists and hepatologists might help elucidate the natural history and long-term prognosis of T2DM-associated NAFLD and establish a standard-of-care guideline for optimal management. We believe that our real-world study may be of use in designing further collaborative studies on the role of SGLT2is in such patients.

In this study, we found that male sex and younger age group showed more favorable results with SGLT2is. In a recent study on patients with T2DM, empagliflozin treatment decreased liver fat content in males but not in females, although the interaction of sex and treatment was not significant (13). In a randomized, active‐controlled trial on patients with T2DM and NAFLD, 5 mg dapagliflozin treatment for 24 weeks improved the controlled attenuation parameter, which was significantly correlated with younger age (18). In another study on patients with T2DM and NAFLD, younger age was associated with a greater reduction in ALT levels with dapagliflozin treatment, but it was not statistically significant (OR = 0.954, *P* = 0.147) (27). Thus, males and younger age people are likely to respond to the SGLT2i therapy for fatty liver. Additional targeted randomized controlled trials focusing on age and sex are required to confirm this finding.

We also found that the advantage of SGLT2i in the improvement of ALT levels was more prominent in patients with baseline BMI > 26 kg/m^2^ and with less weight reduction during follow-up. To our knowledge, the effect of SGLT2i therapy on liver enzyme activities relative to BMI has not been reported in previous studies. This finding warrants mechanistic explanation through further studies and may provide some clue to the mechanism of action of SGLT2i on fatty liver. Since the effect on glucose control was similar between SGLT2i and other OADs (**Table 2**), SGLT2is may improve adipose tissue-induced hepatic inflammation (38) and oxidative stress (39) before weight reduction is achieved. Of note, the effect of non-pharmacologic intervention of body weight reduction might have overshadowed the weight-reducing effect of SGLT2i therapy.

Many studies have discovered a close association between NAFLD and impaired glucose regulation, resulting in the development of T2DM (40). Recent studies provide much evidence for an association between NAFLD and atherosclerosis and cardiovascular diseases (CVD) (41). Of note, the new descriptor ‘metabolic dysfunction-associated fatty liver disease’ (MAFLD) is proposed to replace the term NAFLD, because it more closely implicates obesity and metabolic dysregulation, leading to better identification of individuals with metabolic liver disease (42). MAFLD is not only associated with liver-related complications, but also with adverse cardiometabolic outcomes. Moreover, since many international guidelines recommend using SGLT2is for patients with established atherosclerotic CVD (ASCVD) or chronic kidney disease (CKD) or at high such risk (43, 44), it would be more prudent to conduct randomized controlled trials on people with good glycemic control and no ASCVD or CKD, in order to confirm the magnitude of the beneficial effect of SGLT2is and its mechanism of action.

Our study had several limitations. First, ALT was used as a surrogate marker for NAFLD activity in this study. It has been reported that ALT is a suboptimal marker for diagnosis of NASH with the area under the receiver operating characteristics for ALT level of 0.61–0.62 for NASH (45, 46). However, since ALT response may reflect histologic improvement in NASH (47), transaminase response is suggested as a better endpoint in early phase development trials (48). Apparently, the effect of SGLT2is on NAFLD needs to be further validated by long-term studies with histologic confirmation. Second, since our study was retrospective and observational, our results were prone to selection bias. We have matched most relevant variables by PS to minimize unbalancing, but the potential influence of unmeasured variables might still have biased the outcome. Applying PS matching is likely to achieve a better balance of covariates, but there is no consensus on the best way of capturing all relevant confounders for incorporation into the PS model. PS matching has advantages in situations with large numbers of covariates but relatively few outcome events captured in databases. By contrast, unmatched patients are excluded from analysis, thus increasing validity but at the expense of loss of information and generalizability (49). Moreover, various combinations of OADs have been used in our patients, so the potential interactions of SGLT2is and other OADs could not be fully controlled. These potential sources of bias can only be controlled by further prospective randomized trials. Third, different types of SGLT2is might have affected the outcome differently, but we did not assess the potential difference between individual SGLT2is because of limitations in statistical power given the patient numbers were not large. Fourth, as mentioned above, the hepatic histologic improvement by SGLT2i therapy needs to be assessed by long-term follow-up trials. Finally, since our data were obtained from Korean patients, further validation would be warranted in other ethnic groups.

In conclusion, this PS-matched comparative real-world study showed that the addition of SGLT2is decreased body weight and ALT levels significantly compared with other OADs in T2DM patients with NAFLD on metformin therapy. SGLT2is might be a preferable option for these patients.

## Data Availability Statement

The raw data supporting the conclusions of this article will be made available by the authors, without undue reservation.

## Ethics Statement

The studies involving human participants were reviewed and approved by the institutional review board of Seoul National University Bundang Hospital. Written informed consent for participation was not required for this study in accordance with the national legislation and the institutional requirements.

## Author Contributions

J-WK: designed the research study, analyzed the data and wrote the paper. WE: collected and analyzed the data and wrote the paper. SL: analyzed the data and wrote the paper. All authors contributed to the article and approved the submitted version.

## Funding

This work was supported by a National Research Foundation of Korea (NRF) grant to J-WK, funded by the South Korean Government (2017R1D1A1B03031483). The funders had no role in the study design, data collection and analysis, decision to publish, or preparation of the manuscript.

## Conflict of Interest

The authors declare that the research was conducted in the absence of any commercial or financial relationships that could be construed as a potential conflict of interest.

## References

[1] Lee YH Cho Y Lee BW Park CY Lee DH Cha BS. Nonalcoholic Fatty Liver Disease in Diabetes. Part I: Epidemiology and Diagnosis. Diabetes Metab J (2019) 43:31–45. 10.4093/dmj.2019.0011 30793550 PMC6387876

[2] Anstee QM Targher G Day CP. Progression of NAFLD to Diabetes Mellitus, Cardiovascular Disease or Cirrhosis. Nat Rev Gastroenterol Hepatol (2013) 10:330–44. 10.1038/nrgastro.2013.41 23507799

[3] Hazlehurst JM Woods C Marjot T Cobbold JF Tomlinson JW. Non-Alcoholic Fatty Liver Disease and Diabetes. Metabolism (2016) 65:1096–108. 10.1016/j.metabol.2016.01.001 PMC4943559 26856933

[4] Li Y Liu L Wang B Wang J Chen D. Metformin in Non-Alcoholic Fatty Liver Disease: A Systematic Review and Meta-Analysis. BioMed Rep (2013) 1:57–64. 10.3892/br.2012.18 24648894 PMC3956897

[5] Musso G Cassader M Paschetta E Gambino R. Thiazolidinediones and Advanced Liver Fibrosis in Nonalcoholic Steatohepatitis: A Meta-Analysis. JAMA Intern Med (2017) 177:633–40. 10.1001/jamainternmed.2016.9607 PMC5470366 28241279

[6] He L Liu X Wang L Yang Z. Thiazolidinediones for Nonalcoholic Steatohepatitis: A Meta-Analysis of Randomized Clinical Trials. Med (Baltimore) (2016) 95:e4947. 10.1097/MD.0000000000004947 PMC5079311 27759627

[7] Cui J Philo L Nguyen P Hofflich H Hernandez C Bettencourt R. Sitagliptin *vs.* Placebo for Non-Alcoholic Fatty Liver Disease: A Randomized Controlled Trial. J Hepatol (2016) 65:369–76. 10.1016/j.jhep.2016.04.021 PMC5081213 27151177

[8] Chalasani N Younossi Z Lavine JE Charlton M Cusi K Rinella M. The Diagnosis and Management of Nonalcoholic Fatty Liver Disease: Practice Guidance From the American Association for the Study of Liver Diseases. Hepatology (2018) 67:328–57. 10.1002/hep.29367 28714183

[9] European Association for the Study of The L. European Association for the Study Of D. European Association for the Study Of O. Easl-Easd-Easo Clinical Practice Guidelines for the Management of Non-Alcoholic Fatty Liver Disease. J Hepatol (2016) 64:1388–402. 10.1016/j.jhep.2015.11.004 27062661

[10] Bolinder J Ljunggren O Kullberg J Johansson L Wilding J Langkilde AM. Effects of Dapagliflozin on Body Weight, Total Fat Mass, and Regional Adipose Tissue Distribution in Patients With Type 2 Diabetes Mellitus With Inadequate Glycemic Control on Metformin. J Clin Endocrinol Metab (2012) 97:1020–31. 10.1210/jc.2011-2260 22238392

[11] Sugiyama S Jinnouchi H Kurinami N Hieshima K Yoshida A Jinnouchi K. Dapagliflozin Reduces Fat Mass Without Affecting Muscle Mass in Type 2 Diabetes. J Atheroscler Thromb (2018) 25:467–76. 10.5551/jat.40873 PMC6005223 29225209

[12] Kurinami N Sugiyama S Yoshida A Hieshima K Miyamoto F Kajiwara K. Dapagliflozin Significantly Reduced Liver Fat Accumulation Associated With a Decrease in Abdominal Subcutaneous Fat in Patients With Inadequately Controlled Type 2 Diabetes Mellitus. Diabetes Res Clin Pract (2018) 142:254–63. 10.1016/j.diabres.2018.05.017 29859912

[13] Kahl S Gancheva S Strassburger K Herder C Machann J Katsuyama H. Empagliflozin Effectively Lowers Liver Fat Content in Well-Controlled Type 2 Diabetes: A Randomized, Double-Blind, Phase 4, Placebo-Controlled Trial. Diabetes Care (2020) 43:298–305. 10.2337/dc19-0641 31540903

[14] Kuchay MS Krishan S Mishra SK Farooqui KJ Singh MK Wasir JS. Effect of Empagliflozin on Liver Fat in Patients With Type 2 Diabetes and Nonalcoholic Fatty Liver Disease: A Randomized Controlled Trial (E-LIFT Trial). Diabetes Care (2018) 41:1801–8. 10.2337/dc18-0165 29895557

[15] Sumida Y Murotani K Saito M Tamasawa A Osonoi Y Yoneda M. Effect of Luseogliflozin on Hepatic Fat Content in Type 2 Diabetes Patients With Non-Alcoholic Fatty Liver Disease: A Prospective, Single-Arm Trial (LEAD Trial). Hepatol Res (2019) 49:64–71. 10.1111/hepr.13236 30051943

[16] Latva-Rasku A Honka MJ Kullberg J Mononen N Lehtimaki T Saltevo J. The Sglt2 Inhibitor Dapagliflozin Reduces Liver Fat But Does Not Affect Tissue Insulin Sensitivity: A Randomized, Double-Blind, Placebo-Controlled Study With 8-Week Treatment in Type 2 Diabetes Patients. Diabetes Care (2019) 42:931–7. 10.2337/dc18-1569 30885955

[17] Seko Y Sumida Y Tanaka S Mori K Taketani H Ishiba H. Effect of Sodium Glucose Cotransporter 2 Inhibitor on Liver Function Tests in Japanese Patients With Non-Alcoholic Fatty Liver Disease and Type 2 Diabetes Mellitus. Hepatol Res (2017) 47:1072–8. 10.1111/hepr.12834 27925353

[18] Shimizu M Suzuki K Kato K Jojima T Iijima T Murohisa T. Evaluation of the Effects of Dapagliflozin, a Sodium-Glucose Co-Transporter-2 Inhibitor, on Hepatic Steatosis and Fibrosis Using Transient Elastography in Patients With Type 2 Diabetes and Non-Alcoholic Fatty Liver Disease. Diabetes Obes Metab (2019) 21:285–92. 10.1111/dom.13520 30178600

[19] Eriksson JW Lundkvist P Jansson PA Johansson L Kvarnstrom M Moris L. Effects of Dapagliflozin and N-3 Carboxylic Acids on Non-Alcoholic Fatty Liver Disease in People With Type 2 Diabetes: A Double-Blind Randomised Placebo-Controlled Study. Diabetologia (2018) 61:1923–34. 10.1007/s00125-018-4675-2 PMC6096619 29971527

[20] Ajmera V Belt P Wilson LA Gill RM Loomba R Kleiner DE. Among Patients With Nonalcoholic Fatty Liver Disease, Modest Alcohol Use Is Associated With Less Improvement in Histologic Steatosis and Steatohepatitis. Clin Gastroenterol Hepatol (2018) 16:1511–20.e1515. 10.1016/j.cgh.2018.01.026 29378307 PMC6098737

[21] Yoo S Lee KH Lee HJ Ha K Lim C Chin HJ. Seoul National University Bundang Hospital’s Electronic System for Total Care. Healthc Inform Res (2012) 18:145–52. 10.4258/hir.2012.18.2.145 PMC3402557 22844650

[22] Saadeh S Younossi ZM Remer EM Gramlich T Ong JP Hurley M. The Utility of Radiological Imaging in Nonalcoholic Fatty Liver Disease. Gastroenterology (2002) 123:745–50. 10.1053/gast.2002.35354 12198701

[23] Lee SS Park SH. Radiologic Evaluation of Nonalcoholic Fatty Liver Disease. World J Gastroenterol (2014) 20:7392–402. 10.3748/wjg.v20.i23.7392 PMC4064084 24966609

[24] Lee JH Kim D Kim HJ Lee CH Yang JI Kim W. Hepatic Steatosis Index: A Simple Screening Tool Reflecting Nonalcoholic Fatty Liver Disease. Dig Liver Dis (2010) 42:503–8. 10.1016/j.dld.2009.08.002 19766548

[25] Inoue M Hayashi A Taguchi T Arai R Sasaki S Takano K. Effects of Canagliflozin on Body Composition and Hepatic Fat Content in Type 2 Diabetes Patients With Non-Alcoholic Fatty Liver Disease. J Diabetes Investig (2019) 10:1004–11. 10.1111/jdi.12980 PMC6626966 30461221

[26] Shibuya T Fushimi N Kawai M Yoshida Y Hachiya H Ito S. Luseogliflozin Improves Liver Fat Deposition Compared to Metformin in Type 2 Diabetes Patients With Non-Alcoholic Fatty Liver Disease: A Prospective Randomized Controlled Pilot Study. Diabetes Obes Metab (2018) 20:438–42. 10.1111/dom.13061 28719078

[27] Choi DH Jung CH Mok JO Kim CH Kang SK Kim BY. Effect of Dapagliflozin on Alanine Aminotransferase Improvement in Type 2 Diabetes Mellitus With Non-Alcoholic Fatty Liver Disease. Endocrinol Metab (Seoul) (2018) 33:387–94. 10.3803/EnM.2018.33.3.387 PMC6145967 30229578

[28] Ito D Shimizu S Inoue K Saito D Yanagisawa M Inukai K. Comparison of Ipragliflozin and Pioglitazone Effects on Nonalcoholic Fatty Liver Disease in Patients With Type 2 Diabetes: A Randomized, 24-Week, Open-Label, Active-Controlled Trial. Diabetes Care (2017) 40:1364–72. 10.2337/dc17-0518 28751548

[29] Wong VW Adams LA De Ledinghen V Wong GL Sookoian S. Noninvasive Biomarkers in NAFLD and NASH - Current Progress and Future Promise. Nat Rev Gastroenterol Hepatol (2018) 15:461–78. 10.1038/s41575-018-0014-9 29844588

[30] Kleiner DE Brunt EM Wilson LA Behling C Guy C Contos M. Association of Histologic Disease Activity With Progression of Nonalcoholic Fatty Liver Disease. JAMA Netw Open (2019) 2:e1912565. 10.1001/jamanetworkopen.2019.12565 31584681 PMC6784786

[31] Tang L Wu Y Tian M Sjostrom CD Johansson U Peng XR. Dapagliflozin Slows the Progression of the Renal and Liver Fibrosis Associated With Type 2 Diabetes. Am J Physiol Endocrinol Metab (2017) 313:E563–76. 10.1152/ajpendo.00086.2017

[32] Jojima T Tomotsune T Iijima T Akimoto K Suzuki K Aso Y. Empagliflozin (An SGLT2 Inhibitor), Alone or in Combination With Linagliptin (A DPP-4 Inhibitor), Prevents Steatohepatitis in a Novel Mouse Model of Non-Alcoholic Steatohepatitis and Diabetes. Diabetol Metab Syndr (2016) 8:45. 10.1186/s13098-016-0169-x 27462372 PMC4960737

[33] Akuta N Kawamura Y Watanabe C Nishimura A Okubo M Mori Y. Impact of Sodium Glucose Cotransporter 2 Inhibitor on Histological Features and Glucose Metabolism of Non-Alcoholic Fatty Liver Disease Complicated by Diabetes Mellitus. Hepatol Res (2019) 49:531–9. 10.1111/hepr.13304 30577089

[34] Takeda A Irahara A Nakano A Takata E Koketsu Y Kimata K. The Improvement of the Hepatic Histological Findings in a Patient With Non-Alcoholic Steatohepatitis With Type 2 Diabetes After the Administration of the Sodium-Glucose Cotransporter 2 Inhibitor Ipragliflozin. Intern Med (2017) 56:2739–44. 10.2169/internalmedicine.8754-16 PMC5675935 28924123

[35] Fujimori N Tanaka N Kimura T Sano K Horiuchi A Kato N. Long-Term Luseogliflozin Therapy Improves Histological Activity of Non-Alcoholic Steatohepatitis Accompanied by Type 2 Diabetes Mellitus. Clin J Gastroenterol (2020) 13:83–9. 10.1007/s12328-019-01018-1 31292843

[36] American Diabetes A. 9. Pharmacologic Approaches to Glycemic Treatment: Standards of Medical Care in Diabetes-2020. Diabetes Care (2020) 43:S98–S110. 10.2337/dc20-S009 31862752

[37] Kim MK Ko SH Kim BY Kang ES Noh J Kim SK. K.D.A. 2019 Clinical Practice Guidelines for Type 2 Diabetes Mellitus in Korea. Diabetes Metab J (2019) 43:398–406. 10.4093/dmj.2019.0137 31441247 PMC6712226

[38] Bonnet F Scheen AJ. Effects of SGLT2 Inhibitors on Systemic and Tissue Low-Grade Inflammation: The Potential Contribution to Diabetes Complications and Cardiovascular Disease. Diabetes Metab (2018) 44:457–64. 10.1016/j.diabet.2018.09.005 30266577

[39] Tahara A Kurosaki E Yokono M Yamajuku D Kihara R Hayashizaki Y. Effects of SGLT2 Selective Inhibitor Ipragliflozin on Hyperglycemia, Hyperlipidemia, Hepatic Steatosis, Oxidative Stress, Inflammation, and Obesity in Type 2 Diabetic Mice. Eur J Pharmacol (2013) 715:246–55. 10.1016/j.ejphar.2013.05.014 23707905

[40] Lim S Oh TJ Koh KK. Mechanistic Link Between Nonalcoholic Fatty Liver Disease and Cardiometabolic Disorders. Int J Cardiol (2015) 201:408–14. 10.1016/j.ijcard.2015.08.107 26310987

[41] Lim S Taskinen MR Boren J. Crosstalk Between Nonalcoholic Fatty Liver Disease and Cardiometabolic Syndrome. Obes Rev (2019) 20:599–611. 10.1111/obr.12820 30589487

[42] Lim S Kim JW Targher G. Links Between Metabolic Syndrome and Metabolic Dysfunction-Associated Fatty Liver Disease. Trends Endocrinol Metab (2021) S1043-2760(21):00089–8. 10.1016/j.tem.2021.04.008 33975804

[43] American Diabetes Association. 9. Pharmacologic Approaches to Glycemic Treatment: Standards of Medical Care in Diabetes-2021. Diabetes Care (2021) 44:S111–24. 10.2337/dc21-S009 33298420

[44] Lim S Eckel RH Koh KK. Clinical Implications of Current Cardiovascular Outcome Trials With Sodium Glucose Cotransporter-2 (SGLT2) Inhibitors. Atherosclerosis (2018) 272:33–40. 10.1016/j.atherosclerosis.2018.03.013 29547706

[45] Verma S Jensen D Hart J Mohanty SR. Predictive Value of ALT Levels for non-Alcoholic Steatohepatitis (NASH) and Advanced Fibrosis in Non-Alcoholic Fatty Liver Disease (NAFLD). Liver Int (2013) 33:1398–405. 10.1111/liv.12226 23763360

[46] Balakrishnan M Loomba R. The Role of Noninvasive Tests for Differentiating NASH From NAFL and Diagnosing Advanced Fibrosis Among Patients With NAFLD. J Clin Gastroenterol (2020) 54:107–13. 10.1097/MCG.0000000000001284 PMC7945957 31789757

[47] Hoofnagle JH Van Natta ML Kleiner DE Clark JM Kowdley KV Loomba R. Vitamin E and Changes in Serum Alanine Aminotransferase Levels in Patients With Non-Alcoholic Steatohepatitis. Aliment Pharmacol Ther (2013) 38:134–43. 10.1111/apt.12352 PMC3775262 23718573

[48] Rinella ME Tacke F Sanyal AJ Anstee QM, Participants of the A.E.W. Report on the AASLD/EASL Joint Workshop on Clinical Trial Endpoints in NAFLD. J Hepatol (2019) 71:823–33. 10.1016/j.jhep.2019.04.019 31300231

[49] Loke YK Mattishent K. Propensity Score Methods in Real-World Epidemiology: A Practical Guide for First-Time Users. Diabetes Obes Metab (2020) 22(Suppl 3):13–20. 10.1111/dom.13926 32250525

