# Supplementary material for: Sodium-Glucose Cotransporter-2 Inhibitors Ameliorate Liver Enzyme Abnormalities in Korean Patients With Type 2 Diabetes Mellitus and Nonalcoholic Fatty Liver Disease
Source: Front Endocrinol (Lausanne). 2021 Jun 10;12:613389. doi: 10.3389/fendo.2021.613389 (PMC8222919; doi:10.3389/fendo.2021.613389)
Supplement: Supplementary file 1 [file Table_1.docx]

| **Supplementary Table 1.** Comparison of 3-, 6-, and 9-month changes in transaminase levels between the propensity score-matched DPP4i and SGLT2i groups | | | |
| --- | --- | --- | --- |
|  | DPP4i  n = 160 | SGLT2i  n = 95 | *P* |
| AST changes (IU/L) |  |  |  |
| 3 months | –3 (–6, 0) | –7 (–12, –1) | 0.182 |
| 6 months | –4 (–7, 0) | –7 (–13, –1) | 0.299 |
| 9 months | –3 (–6, 0) | –8 (–14, –2) | 0.115 |
| ALT changes (IU/L) |  |  |  |
| 3 months | –4 (–8, 0) | –11 (–18, –4) | 0.068 |
| 6 months | –4 (–8, 0) | –13 (–20, –6) | 0.023 |
| 9 months | –3 (–8, 1) | –15 (–22, –7) | 0.009 |
| Values are expressed as the mean (95% confidence interval). *P* values were calculated using Student’s *t*-test. DPP4i, dipeptidyl peptidase 4 inhibitor; ALT, alanine aminotransferase; AST, aspartate aminotransferase. | | | |
